# Case report: Analysis of phage therapy failure in a patient with a *Pseudomonas aeruginosa* prosthetic vascular graft infection

**DOI:** 10.3389/fmed.2023.1199657

**Published:** 2023-05-19

**Authors:** Lucia Blasco, Inmaculada López-Hernández, Miguel Rodríguez-Fernández, Javier Pérez-Florido, Carlos S. Casimiro-Soriguer, Sarah Djebara, Maya Merabishvili, Jean-Paul Pirnay, Jesús Rodríguez-Baño, María Tomás, Luis Eduardo López Cortés

**Affiliations:** ^1^Translational and Multidisciplinary Microbiology (MicroTM)-Biomedical Research Institute (INIBIC), University of A Coruña (UDC), A Coruña, Spain; ^2^Microbiology Service, A Coruña Hospital (HUAC), University of A Coruña (UDC), A Coruña, Spain; ^3^Unidad Clínica de Enfermedades Infecciosas y Microbiología, Hospital Universitario Virgen Macarena, Seville, Spain; ^4^Departamentos de Medicina y Microbiología, Facultad de Medicina, Universidad de Sevilla, Seville, Spain; ^5^Instituto de Biomedicina de Sevilla (IBiS)/CSIC, Seville, Spain; ^6^CIBERINFEC, Instituto de Salud Carlos III, Madrid, Spain; ^7^Unit of Infectious Diseases and Microbiology, Valme University Hospital, Institute of Biomedicine of Sevilla, Seville, Spain; ^8^Computational Medicine Platform, Andalusian Public Foundation Progress and Health-FPS, Seville, Spain; ^9^Computational Systems Medicine, Institute of Biomedicine of Seville, IBiS, University Hospital Virgen del Rocío/CSIC/University of Sevilla, Seville, Spain; ^10^Laboratory for Molecular and Cellular Technology, Queen Astrid Military Hospital, Neder-over-Heembeek, Belgium

**Keywords:** phage, phage therapy, antibiotic resistance, *Pseudomonas aeruginosa*, bypass, prosthetic vascular graft infection

## Abstract

Clinical case of a patient with a *Pseudomonas aeruginosa* multidrug-resistant prosthetic vascular graft infection which was treated with a cocktail of phages (PT07, 14/01, and PNM) in combination with ceftazidime-avibactam (CZA). After the application of the phage treatment and in absence of antimicrobial therapy, a new *P. aeruginosa* bloodstream infection (BSI) with a septic residual limb metastasis occurred, now involving a wild-type strain being susceptible to ß-lactams and quinolones. Clinical strains were analyzed by microbiology and whole genome sequencing techniques. In relation with phage administration, the clinical isolates of *P. aeruginosa* before phage therapy (HE2011471) and post phage therapy (HE2105886) showed a clonal relationship but with important genomic changes which could be involved in the resistance to this therapy. Finally, phenotypic studies showed a decrease in Minimum Inhibitory Concentration (MIC) to ß-lactams and quinolones as well as an increase of the biofilm production and phage resistant mutants in the clinical isolate of *P. aeruginosa* post phage therapy.

## Introduction

Prosthetic vascular graft infections (PVGI) are complicated events associated with high morbidity and mortality rates. PVGI incidence is between 1 and 6%, showing a death rate range between 15 to 75% with a rate of major amputation that may reach 70%, which is especially caused by aortic graft (1). PVGI are usually caused by the more virulent microorganisms, such as *Staphylococcus aureus, Escherichia coli, Pseudomonas aeruginosa, Klebsiella* spp, *Proteus* spp., and *Enterobacter* spp. (2). Carbapenem-resistant *Pseudomonas aeruginosa* is one the most critical pathogens according to the World Health Organization (WHO) and poses a particular threat in hospitals, nursing homes, and among patients whose care requires devices such as ventilators and blood catheters (3). Several mechanisms of resistance to carbapenems have been described in clinical isolates of *Pseudomonas aeruginosa*, among them, we can highlight -lactamases, mutations in porins, and overexpression of efflux pumps (4).

Phage therapy is a promising new treatment against infections produced by multi-drug resistant pathogens (5). To improve phage therapy application, it would be necessary to know more about the clinical response and bacterial host-phage interactions. The monitoring of these interactions can be done by massive sequencing, thus identifying the genes affected by mutations that occur during therapy, and therefore directing the way phage therapy should be applied.

In this work, we analyzed the clinical, microbiological, and molecular features of *P. aeruginosa* isolates in a case of prosthetic vascular graft infection (PVGI) after phage administration was deemed unsuccessful. This knowledge could allow the development of strategies to improve the use of clinical use of phage therapy.

## Case report

**Figure 1:**
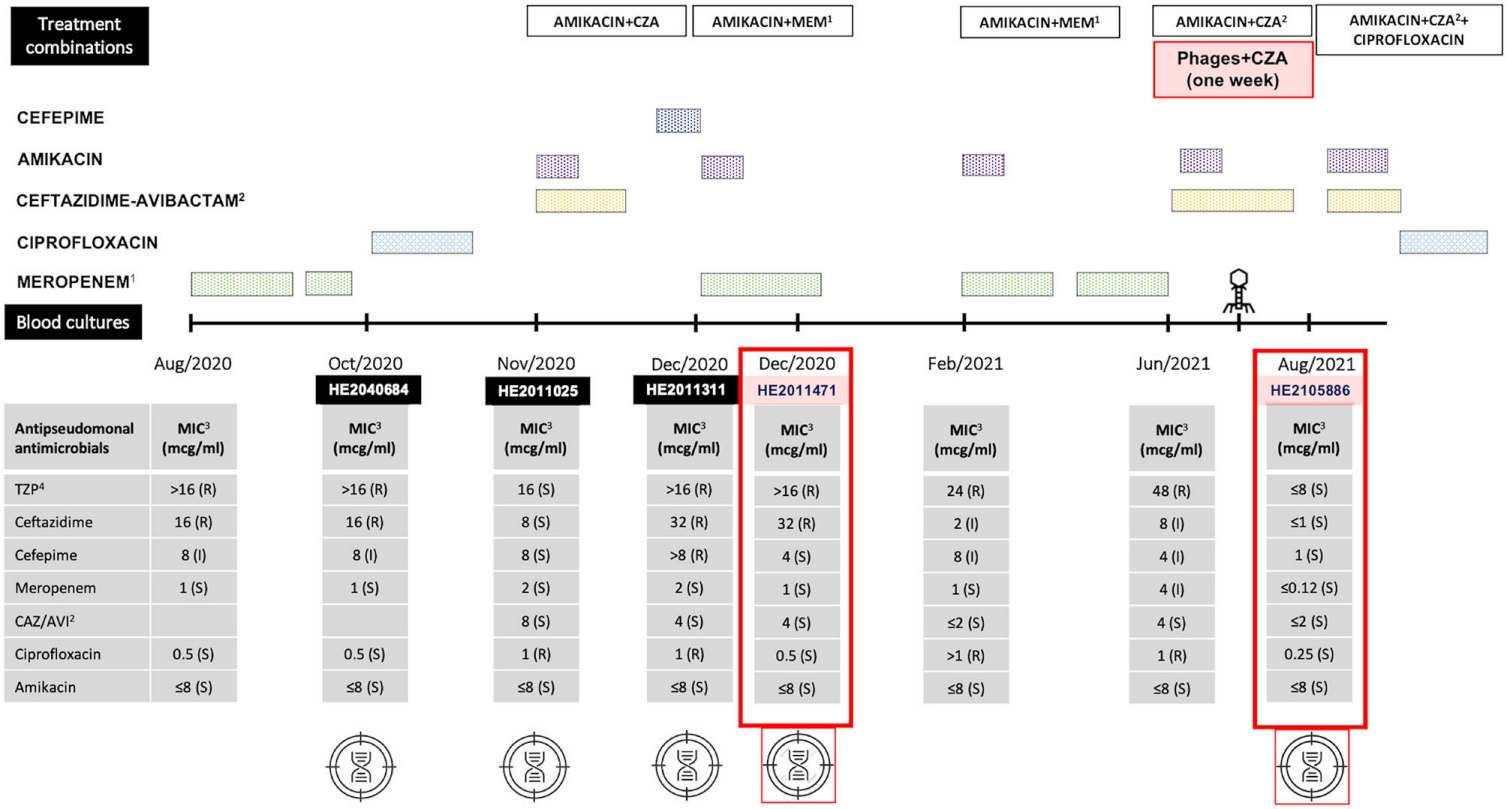
Timeline depicting the different antibiotic and phage therapies, the isolation of *Pseudomonas aeruginosa* strains from blood cultures and their antibiotic susceptibility and genomic analyses. PFGE of these strains in described in Supplementary Figure S1. Antimicrobial information: ^1^MEM, Meropenem. ^2^CZA, Ceftazidime-avibactam. ^3^MIC, Minimum inhibitory concentration. ^4^TZP, Piperacillin-tazobactam. The five isolates that underwent Whole Genome Sequencing (WGS) are shown with the “target” symbols; of those, the two isolates that are further analyzed in the manuscript, *P. aeruginosa* isolate HE2011471 (pre-phage therapy) and *P. aeruginosa* isolate HE2105886 (post-phage therapy) are highlighted with the red boxes.

This work presents the clinical case of a man in his fifties who developed recurrent bloodstream infections (BSI) caused by ceftazidime and piperacillin-tazobactam resistant *Pseudomonas aeruginosa*. In August 2020, an axillo-bifemoral bypass was performed in this patient because of a severe infra-renal aorta atherosclerotic occlusion (Leriche syndrome). Shortly after, the patient first developed a BSI, which was interpreted as secondary to an infected sacral ulcer, and was treated with meropenem at dose of 1 g every 8 h for 17 days. From November to June 2021, the patient suffered several recurrences of BSI due to *P. aeruginosa*. After the second relapse, ^18^F-fluorodeoxyglucose (^18^F-FDG) PET/CT was performed, showing radioactive-labeled glucose uptake along the pre-clavicular graft region (SUVmax value, 5.29), suggesting prosthetic vascular graft infection (PVGI). Graft replacement was rejected due to high risk of contralateral leg ischemia and bypass thrombosis if partial bypass replacement was done. The patient subsequently received different targeted antibiotherapies for 2 to 6 weeks (Figure 1). Two new *P. aeruginosa* BSI relapses occurred between March and May 2021. Ceftolozane-tazobactam was not used (not available), and off-label tebipenem use was denied; treatment was therefore as shown in Figure 1. Simultaneously, therapeutic phages were obtained from the Queen Astrid Military Hospital (Brussels, Belgium) in view of a possible compassionate use of phage therapy.

Phage susceptibility of the clinical isolate was determined in the A Coruña University Hospital, and three *P. aeruginosa* phages were selected. Two (14/01 and PT07) were myoviruses, and one (PNM) was a podovirus. Two of the phages had known receptors, being lipopolysaccharide (LPS) for phage 14/01, and the type IV pili for PNM (6, 7). In July 2021, 70 ml of the bacteriophage cocktail, consisting of the three phages, each at a concentration of 10^7^ plaque forming units (PFU)/mL for a total dose of 2.1 × 10^9^ PFU/day, was administered intravenously, once a day in a 6-h infusion for 3 days in an inpatient regimen. Thereafter, and for four additional days, this cocktail of phages alongside ceftazidime-avibactam were administered by outpatient parenteral antimicrobial therapy (OPAT) at the patient's request. In summary, phage therapy was used for 1 week, with ceftazidime-avibactam being used for 8 weeks (from 6 weeks before, to 2 weeks after phage therapy). Importantly, no adverse events were observed.

Ceftazidime-avibactam was applied for 8 weeks (from 6 weeks before to 2 weeks after the phage therapy). In August 2021, in the absence of antimicrobial therapy, a new *P. aeruginosa* BSI with a septic residual limb metastasis occurred, now involving a wild-type strain being susceptible to ß-lactams and quinolones. Finally, upon a multidisciplinary discussion, a proximal vascular prosthesis replacement combined with antibiotherapy in OPAT was performed. As of this writing, 2023, after 10 months without treatment, the patient remains asymptomatic.

**Figure 2:**
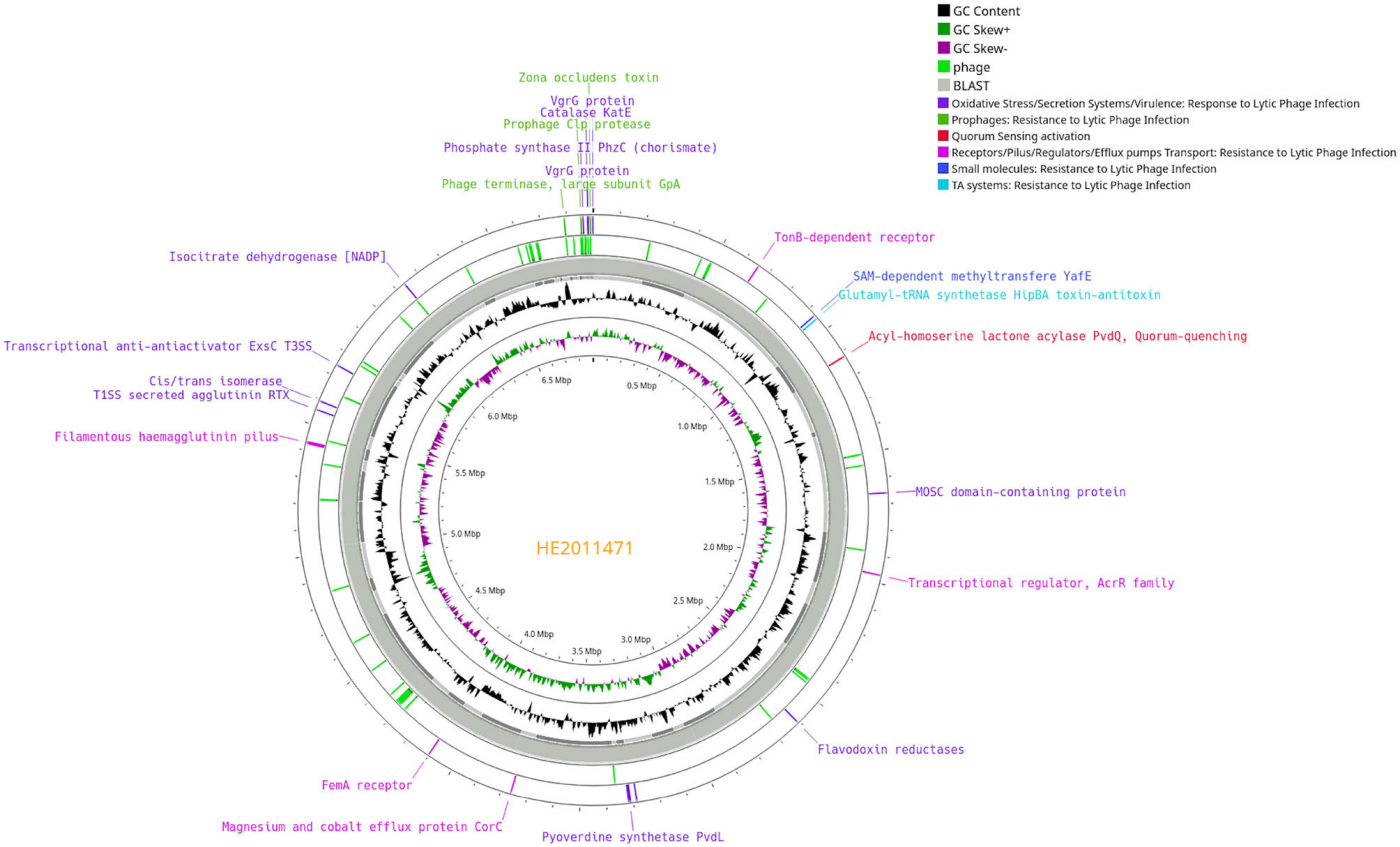
Circular chromosomic view of the bacterial genomes of two *Pseudomonas aeruginosa* isolates: *P. aeruginosa* isolate HE2105886 (after phage treatment) in comparison with *P. aeruginosa* isolate HE2011471 (prior to phage administration). The SNPs found in *P. aeruginosa* isolate HE2105886 respect to *P. aeruginosa* isolate HE2011471 are indicated.

**Figure 3:**
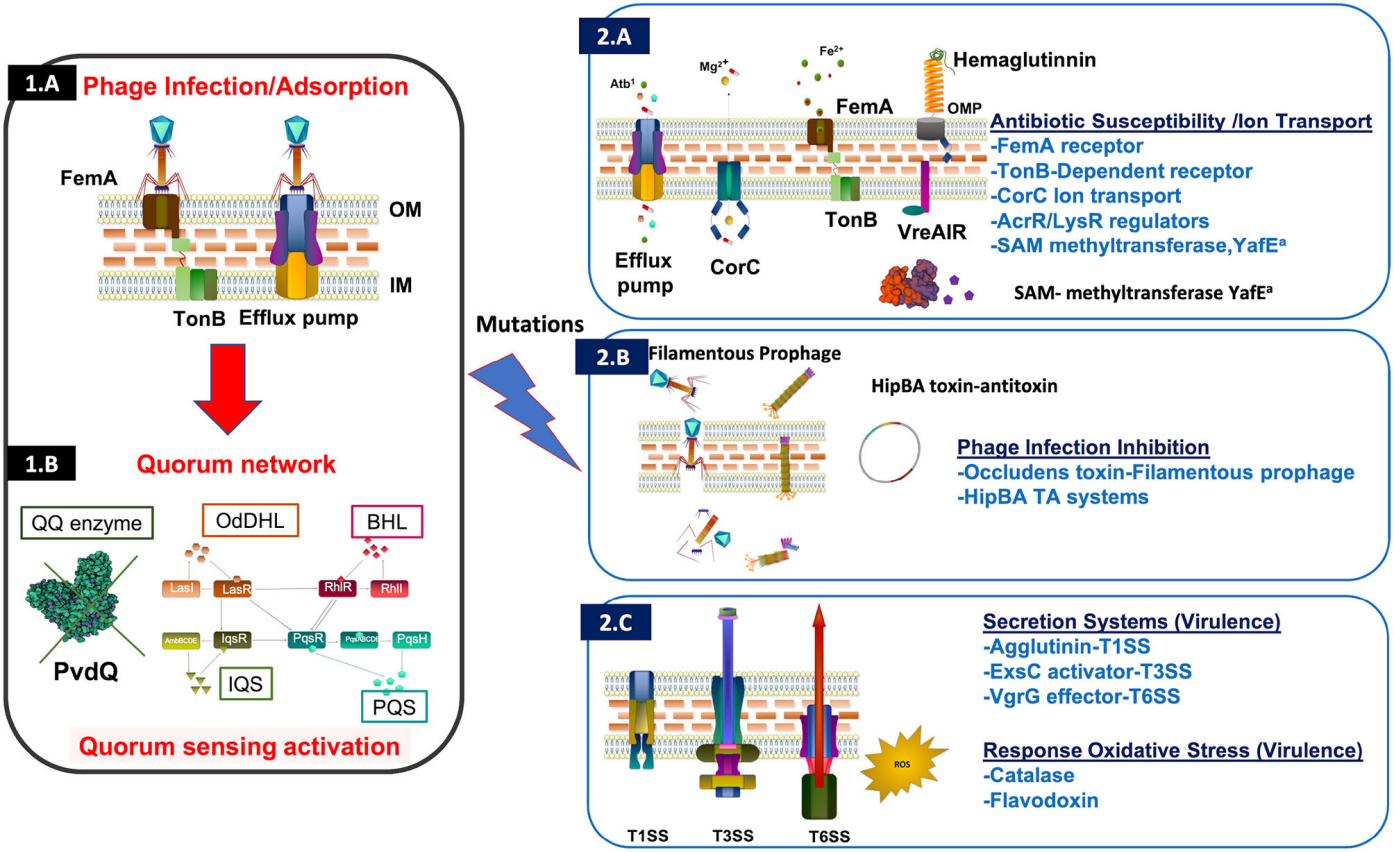
Graphic representation of the proteins coded by the *P. aeruginosa* HE2105886 genome (post-phage therapy isolate), that presented mutations when compared to pre-phage therapy isolate, with emphasis of changes associated with mechanisms of resistance and response to phage infection (12). Among them we highlight phage infection by adsorption as well as Quorum Sensing activation **(1A, 1B)** alteration of the antibiotic susceptibility (receptors and efflux pumps) **(2A)**, inhibition of the phage infection (filamentous prophages, TA (toxin-antitoxin) systems and small molecules) (13–15) **(2B)** and finally, virulence (oxidative stress and secretion systems) **(2C)**. ^a^Small molecules such as aminoglycosides which participate in the inhibition of the phage infection well as bacterial antimicrobial profile.

Eight blood culture isolates obtained between November 2020 and August 2021 were characterized by microbiological analysis. In August 2021, these isolates were identified by matrix assisted laser desorption/ionization—time of flight mass spectrometry (MALDI-TOF MS, Bruker Daltonics) as *P. aeruginosa*. Antimicrobial susceptibility testing was performed by broth microdilution (Microscan, Beckman Coulter) and interpreted using European Committee on Antimicrobial Susceptibility Testing (EUCAST) criteria. Pulsed-field gel electrophoresis (PFGE) revealed that four representative isolates, from 2020 and 2021, showed the same pattern and therefore were clonal (Supplementary Figure S1). Genomic sequence of four representative isolates showed that these isolates belonged to the high-risk clone ST308 with core genome MLST (MLST)cgST2675. High-risk clones are those with a wide dissemination and a global spread associated with a multidrug resistant (MDR) or extensively drug resistant (XDR) profiles including extended-spectrum β-lactamases (ESBLs) and carbapenemases (8). A search for antimicrobial resistance genes in the ResFinder database did not identify any acquired resistance genes such as ß-lactamases (9). Analysis of 40 chromosomal resistance genes revealed natural polymorphisms (SNPs) in numerous genes, including some changes with unknown effect (10). The profile of all isolates was identical for all genes studied, except for the *nfxB* gene, a transcriptional repressor that regulates the efflux pump MexCD-OprJ (11), in which multiple amino acid changes were observed in the last 3 isolates (Supplementary Table S1). Moreover, comparison of the genomes of *P. aeruginosa* isolates HE2011471 (previous to phage administration) and HE2105886 (1 month after phage treatment) revealed important mutations (Figure 2). Interestingly, several detected genomic changes could be involved in phage resistance (Supplementary Table S2, Figure 3). *P. aeruginosa* isolate HE2105886, recovered 1 month after phage treatment, exhibited a decrease in the Minimum Inhibitory Concentration (MICs) of ß-lactam and quinolone antibiotics (Figure 1) probably due to the mutations of the membrane receptors, regulators and efflux pumps. However, we did not test any of these isolates against the individual phages in the cocktail to track the sensitivity or resistance to each of them.

**Figure 4:**
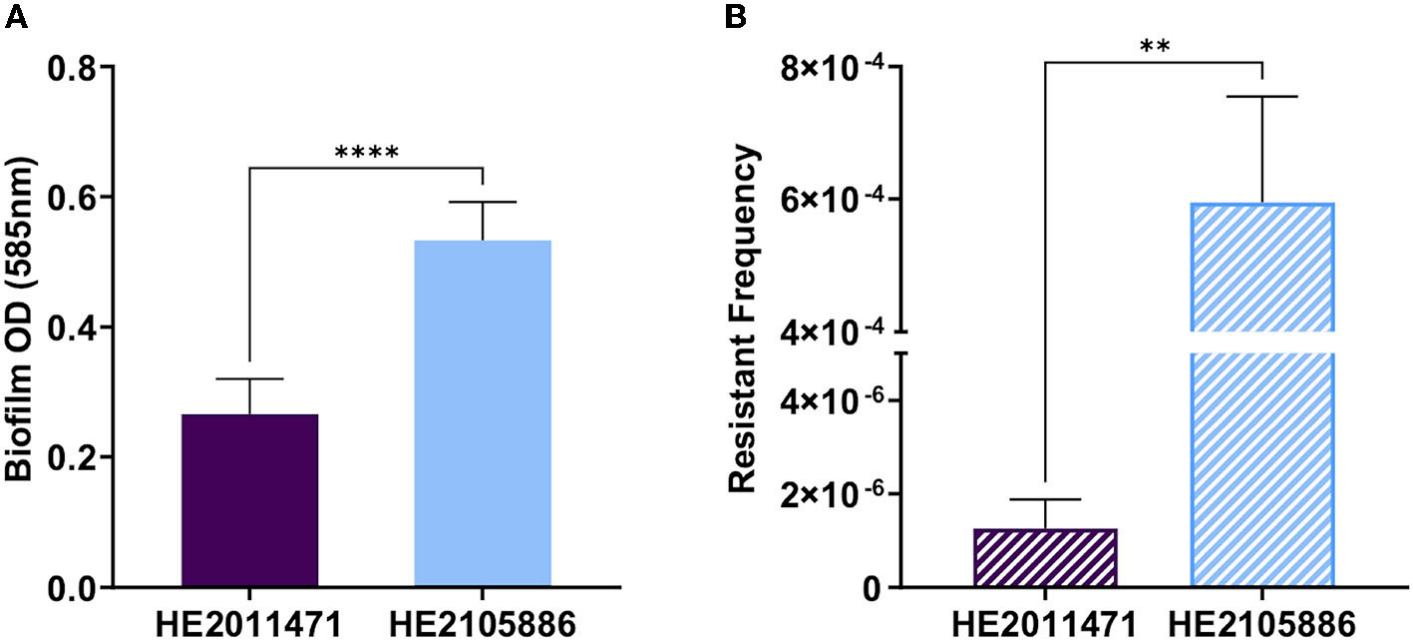
Comparison of the biofilm production **(A)** and the frequency of occurrence of phage resistant mutants **(B)** between the *Pseudomonas aeruginosa* isolates HE2011471 (pre-phage therapy) and HE2105886 (post-phage therapy). Statistical analysis is *t*-test with Graphpad 9.0.0 ^****^<0.0001; **0.0030.

Finally, the post-phage therapy isolate exhibited increased production of biofilm and phage-resistant mutants. These two tests were deemed relevant, as bacteria can form biofilms as a response to environmental stress, such as the presence of antibiotics, and this, alongside the emergence of phage-resistance could explain the observed failure to eradicate the infection with phage therapy (Figures 4A, B) (16).

## Discussion

This case highlights three key issues. The first relates to the use of combined therapy (antibiotics and phages) vs. monotherapy (antibiotic or phages) as a definitive treatment for *P. aeruginosa* BSI. So far, combination therapy has not been associated with reduced mortality or any advantages in terms of clinical outcome or successful treatment of recurrent/persistent bacteraemia (17). The second issue concerns prosthetic vascular graft infection (PVGI) diagnosis and its best management. At the early stages of PVGI, a high degree of suspicion is essential. Indium-111-labeled white blood cell scintigraphy plus single-photon emission computed tomography (SPECT/CT) could reduce the false positive rates observed with PET/CT (18). In the present case, PET/CT was not performed at the early postoperative stage, because of the possibility of a false positive result. Regarding the treatment, graft excision is the preferred surgical approach. However, some patients are considered unacceptable surgical candidates due to underlying comorbidities or technically unfeasible surgery. When this occurs, lifelong suppressive antimicrobial therapy is an option, but is not free of side-effects or the risk of development of antimicrobial resistance. As the patient was initially denied surgical treatment, long-term suppressive treatment with quinolones was administered. However, ciprofloxacin-resistance developed, and surgery was finally deemed key to a favorable outcome. Furthermore, there is no evidence-based recommendation for either PVGI antimicrobial treatment or its optimal duration; minimum intravenous therapy for 6 weeks followed by oral antibiotherapy for up to 6 months has been proposed (19, 20). ^18^F-FDG PET/CT-guided treatment duration seems feasible and would allow treatment to be tailored to individual patients (21). The third highlighted issue is related to phage therapy. Phage therapy involves the targeted application of strictly virulent phages that can specifically infect and lyse the targeted pathogenic bacteria they encounter, hereby releasing virion progeny that continues the lytic cycle. A major advantage of phages is the minimal impact on non-target bacteria or body tissues (22). A recent systematic review suggested that phage therapy is safe and may be effective in different difficult-to-treat infections (23). Interestingly, a previously-reported case of ß-lactam resistance in an MDR *P. aeruginosa* isolate causing an aortic graft infection, showed the reversion of the infection after treatment with a phage whose receptor was the outer membrane protein M (OprM) of the MexAB- and MexXY-multidrug efflux pumps, associated with antibiotic resistance and which was no longer expressed in the selected phage-resistant bacterial strain (24). In the present case, even though phage therapy did not cure the infection, the posterior infection recurrence was caused by an antibiotic susceptible isolate that belonged to the same lineage as the one that was causing the pre-phage treatment episodes of infection. However, the recurrent isolate was recovered 1 month after the phage therapy, and it is possible that the resensitization (to -lactams and quinolones) could have been due to phage action which produced genomic changes in the membrane receptors, regulators and efflux pumps (Supplementary Table S2) or to a spontaneous evolution. In order to confirm it, further studies could be carried out as to demonstrate that these mutations located in the membrane receptors, regulators and efflux pumps proteins indeed render a strain phage-resistant and susceptible to antimicrobials. These studies would involve constructing several plasmids encoding the wild-type genes with lack of the mutations and overexpressing them in the *P. aeruginosa* isolate HE2105886 to restore the phage susceptibility and the resistance to antimicrobials.

One strength of this study is that phage therapy was able to be safely administered in OPAT for greater patient convenience. To our knowledge, this has only been reported once before, in a series of six cases, without phage-related adverse events (25).

The main limitations of our study were: first, we did not perform *in vitro* studies of the effect of the phages in combination with antibiotics, especially ß-lactams; second, we could not study the *P. aeruginosa* clinical isolates during the seven days of application of phage therapy. Finally, it should be noticed that there are no national or local phage banks with characterized phages against *P. aeruginosa* in which specific phages could be selected to provide personalized approach in patients with complex infections caused by these bacteria.

In conclusion, in complex *P. aeruginosa* infections the choice of antibiotic therapy and its duration are crucial for minimizing antibiotic pressure and development of resistance. Although we have not demonstrated that phage treatment was effective in this case, studying the molecular mechanisms of resistance to phages and bacteria-phage interactions are key to improving phage therapy in the near future.

## Material/methods

### Microbiology studies

The isolates were identified by MALDI-TOF MS (Bruker Daltonics). Antimicrobial susceptibility testing was performed by broth microdilution with a Microscan system (Beckman Coulter), and the results were interpreted according to the clinical breakpoints defined by the European Committee on Antimicrobial Susceptibility Testing (EUCAST). PFGE analysis was performed using *Spe*I.

### Genomic sequencing and bioinformatic tools

Whole genome sequencing of the five isolates was performed in a HiSeq 2500 sequencing system (Illumina), and sequences were assembled using SPAdes 3.10.1.

Clonality testing of genomes was carried out using MLST Finder (from CGE, available at https://bitbucket.org/genomicepidemiology/mlst.git) and Ridom SeqSphere+ v8.5, and the resistance mechanisms were analyzed using the ResFinder database. SNP calling was performed using snippy v4.6.0 against the NCBI PGAP annotation (https://www.ncbi.nlm.nih.gov/genome/annotation_prok/). The assemblies were visually inspected using Bandage v0.8.1. (https://rrwick.github.io/Bandage/). Further functional annotation genes and prophage elements were confirmed using Blastx (http://blast.ncbi.nlm.nih.gov), Hhmer (http://hmmer.org), and also the HHpred tool (https://toolkit.tuebingen.mpg.de/tools/hhpred), which predict functions through protein structure.

Assembled genomes of *P. aeruginosa* isolates were analyzed using Phaster (PHAge Search Tool Enhanced Release) software (https://phaster.ca/) and SourceFinder (https://cge.food.dtu.dk/services/SourceFinder/).

### Biofilm production

Overnight cultures of *P. aeruginosa* isolates HE2011471 and HE2105886 were diluted 1:100 and used to inoculate 100 μL of LB broth in a 96 multi-well plate. The plate was incubated for 24 h at 37°C in darkness. The supernatant was discarded and the wells were washed with PBS. One hundred μL of methanol was then added to each well and discarded after 10 min. When the methanol had completely evaporated, 100 μL of crystal violet (0.1%) was added and discarded after 15 min. Finally, the wells were washed with PBS before the addition of 150 μL of acetic acid (30%), and the absorbance was measured at OD 585 nm.

### Frequency of occurrence of phage resistant mutants

The frequency of occurrence of phage resistant mutants was determined as previously described, with some modifications (26). Overnight cultures of isolates HE2011471 and HE 2105886 were diluted 1:100 in LB and grown to an OD600 nm of 0.6–0.7. An aliquot of 100 μL of the culture containing 10^8^ colony forming units (CFU)/mL was serially diluted, and each dilution was mixed with 100 μL of 10^9^ PFU/mL phage cocktail and then plated by the agar overlay method (27). The plates were incubated at 37°C for 18 h and the number of CFUs was counted. The frequency of occurrence of phage resistant mutants and phage resistant mutants was calculated by dividing the number of resistant bacteria by the total number of sensitive bacteria.

## Data availability statement

The datasets presented in this study can be found in online repositories. The names of the repository/repositories and accession number(s) can be found in the article/Supplementary material.

## Ethics statement

Written informed consent was obtained from the patient for the publication of any potentially identifiable images or data included in this article.

## Author contributions

LB, IL-H, MR-F, JP-F, and CC-S conducted the experiments, analyzed the results, and wrote the manuscript. SD, MM, and J-PP revised the results and phage therapy administration. JR-B, MT, and LL obtained the research funding, directed the clinical settings, and supervised the writing of the manuscript. All authors contributed to the article and approved the submitted version.

## Supplementary material

Supplementary Figure S1 PFGE (*Pulsed Field Gel Electrophoresis*) patterns of four sequential *Pseudomonas aeruginosa isolates*.

Supplementary Table S1 Mutations encountered in proteins known to be related to antimicrobial resistance in *Pseudomonas aeruginosa*. The mutations present in *P. aeruginosa* isolate HE2105886 in comparison to *P. aeruginosa* isolate HE2011471 are shown.

Supplementary Table S2 Mutations encountered in proteins probably to be related to phage resistance in response to lytic phage infection in *P. aeruginosa* isolates *P. aeruginosa* isolate HE2105886 in comparison to *P. aeruginosa* isolate HE2011471. ^a^ Proteins located in prophages as identified by Phaster and SourceFinder.

## References

[1] Legout L Sarraz-Bournet B D'Elia PV Devos P Pasquet A Caillaux M. Characteristics and prognosis in patients with prosthetic vascular graft infection: a prospective observational cohort study. Clin Microbiol Infect. (2012) 18:352–8. 10.1111/j.1469-0691.2011.03618.x 21883666

[2] SR BMaK. Infections and Antibiotics in Vascular Surgery. In: Basic Science and Clinical Correlations, editor. White RA and Hollier LH, Philadelphia: J. B. Lippincott company. (1994).

[3] Tacconelli E Carrara E Savoldi A Harbarth S Mendelson M Monnet DL. Discovery, research, and development of new antibiotics: the WHO priority list of antibiotic-resistant bacteria and tuberculosis. Lancet Infect Dis. (2018) 18:318–27. 10.1016/S1473-3099(17)30753-3 29276051

[4] Cabot G Ocampo-Sosa AA Tubau F Macia MD Rodríguez C Moya B. Spanish Network for Research in Infectious Diseases (REIPI). Overexpression of AmpC and efflux pumps in Pseudomonas aeruginosa isolates from bloodstream infections: prevalence and impact on resistance in a Spanish multicenter study. Antimicrob Agents Chemother. (2011) 55:1906–11. 10.1128/AAC.01645-10 21357294 PMC3088238

[5] Hatfull GF Dedrick RM Schooley RT. Phage Therapy for Antibiotic-Resistant Bacterial Infections. Annu Rev Med. (2022) 73:197–211. 10.1146/annurev-med-080219-122208 34428079

[6] Ceyssens PJ Glonti T Kropinski NM Lavigne R Chanishvili N Kulakov L. Phenotypic and genotypic variations within a single bacteriophage species. Virol J. (2011) 8:134. 10.1186/1743-422X-8-134 21429206 PMC3072928

[7] Ceyssens PJ Miroshnikov K Mattheus W Krylov V Robben J Noben JP. Comparative analysis of the widespread and conserved PB1-like viruses infecting *Pseudomonas aeruginosa*. Environ Microbiol. (2009) 11:2874–83. 10.1111/j.1462-2920.2009.02030.x 19678828

[8] Del Barrio-Tofiño E López-Causapé C Oliver A. *Pseudomonas aeruginosa* epidemic high-risk clones and their association with horizontally-acquired β-lactamases: 2020 update. Int J Antimicrob Agents. (2020) 56:106196. 10.1016/j.ijantimicag.2020.106196 33045347

[9] Florensa AF Kaas RS Clausen PTLC Aytan-Aktug D Aarestrup FM. ResFinder - an open online resource for identification of antimicrobial resistance genes in next-generation sequencing data and prediction of phenotypes from genotypes. Microb Genom. (2022) 8:000748. 10.1099/mgen.0.000748 35072601 PMC8914360

[10] Cortes-Lara S Barrio-Tofiño ED López-Causapé C Oliver A GEMARA- SEIMC/REIPI *Pseudomonas* study Group. Predicting Pseudomonas aeruginosa susceptibility phenotypes from whole genome sequence resistome analysis. Clin Microbiol Infect. (2021) 27:1631–7. 10.1016/j.cmi.2021.05.011 34015532

[11] Alcalde-Rico M Olivares-Pacheco J Alvarez-Ortega C Cámara M Martínez JL. Role of the Multidrug Resistance Efflux Pump MexCD-OprJ in the *Pseudomonas aeruginosa* Quorum Sensing Response. Front Microbiol. (2018) 9:2752. 10.3389/fmicb.2018.02752 30532741 PMC6266676

[12] Luthe T Kever L Thormann K Frunzke J. Bacterial multicellular behavior in antiviral defense. Curr Opin Microbiol. (2023) 74:102314. 10.1016/j.mib.2023.102314 37030144

[13] Bleriot I Trastoy R Blasco L Fernández-Cuenca F Ambroa A Fernández-García L. Genomic analysis of 40 prophages located in the genomes of 16 carbapenemase-producing clinical strains of *Klebsiella pneumoniae*. Microb Genom. (2020) 6:e000369. 10.1099/mgen.0.000369 32375972 PMC7371120

[14] Bleriot I Blasco L Pacios O Fernández-García L Ambroa A López M. The role of PemIK (PemK/PemI) type II TA system from *Klebsiella pneumoniae* clinical strains in lytic phage infection. Sci Rep. (2022) 12:4488. 10.1038/s41598-022-08111-5 35296704 PMC8927121

[15] Hardy A Kever L Frunzke J. Antiphage small molecules produced by bacteria-beyond protein-mediated defenses. Trends Microbiol. (2023) 31:92–106. 10.1016/j.tim.2022.08.001 36038409

[16] Castledine M Padfield D Sierocinski P Soria Pascual J Hughes A Mäkinen L. Parallel evolution of *Pseudomonas aeruginosa* phage resistance and virulence loss in response to phage treatment in vivo and in vitro. Elife. (2022) 11:e73679. 10.7554/eLife.73679 35188102 PMC8912922

[17] Babich T Naucler P Valik JK Giske CG Benito N Cardona R. Combination versus monotherapy as definitive treatment for *Pseudomonas aeruginosa* bacteraemia: a multicentre retrospective observational cohort study. J Antimicrob Chemother. (2021) 76:2172–81. 10.1093/jac/dkab134 33993273

[18] Reinders Folmer EI Von Meijenfeldt GCI Van der Laan MJ Glaudemans AWJM Slart RHJA Saleem BR. Diagnostic Imaging in Vascular Graft Infection: A Systematic Review and Meta-Analysis. Eur J Vasc Endovasc Surg. (2018) 56:719–29. 10.1016/j.ejvs.2018.07.010 30122333

[19] Leroy O Meybeck A Sarraz-Bournet B d'Elia P Legout L. Vascular graft infections. Curr Opin Infect Dis. (2012) 25:154–8. 10.1097/QCO.0b013e3283501853 22248976

[20] Wilson WR Bower TC Creager MA Amin-Hanjani S O'Gara PT Lockhart PB. Vascular graft infections, mycotic aneurysms, and endovascular infections: a scientific statement from the american heart association. Circulation. (2016) 134:e412–60. 10.1161/CIR.0000000000000457 27737955

[21] Kung BT Seraj SM Zadeh MZ Rojulpote C Kothekar E Ayubcha C. An update on the role of ^18^F-FDG-PET/CT in major infectious and inflammatory diseases. Am J Nucl Med Mol Imaging. (2019) 9:255–73. 31976156 PMC6971480

[22] Abedon ST Kuhl SJ Blasdel BG Kutter EM. Phage treatment of human infections. Bacteriophage. (2011) 1:66–85. 10.4161/bact.1.2.15845 22334863 PMC3278644

[23] Uyttebroek S Chen B Onsea J Ruythooren F Debaveye Y Devolder D. Safety and efficacy of phage therapy in difficult-to-treat infections: a systematic review. Lancet Infect Dis. (2022) 22:e208–20. 10.1016/S1473-3099(21)00612-5 35248167

[24] Chan BK Turner PE Kim S Mojibian HR Elefteriades JA Narayan D. Phage treatment of an aortic graft infected with *Pseudomonas aeruginosa*. Evol Med Public Health. (2018) 2018:60–6. 10.1093/emph/eoy005 29588855 PMC5842392

[25] Aslam S Lampley E Wooten D Karris M Benson C Strathdee S. Lessons learned from the first 10 consecutive cases of intravenous bacteriophage therapy to treat multidrug-resistant bacterial infections at a single center in the United States. Open Forum Infect Dis. (2020) 7:ofaa389. 10.1093/ofid/ofaa389 33005701 PMC7519779

[26] Blasco L Ambroa A Lopez M Fernandez-Garcia L Bleriot I Trastoy R. Combined Use of the Ab105-2ϕΔCI Lytic Mutant Phage and Different Antibiotics in Clinical Isolates of Multi-Resistant *Acinetobacter baumannii*. Microorganisms. (2019) 7:556. 10.3390/microorganisms7110556 31726694 PMC6921023

[27] Kropinski AM Mazzocco A Waddell TE Lingohr E Johnson RP. Enumeration of bacteriophages by double agar overlay plaque assay. Methods Mol Biol. (2009) 501:69–76. 10.1007/978-1-60327-164-6_7 19066811

