# Supplementary figures and images for: Case report: Analysis of phage therapy failure in a patient with a Pseudomonas aeruginosa prosthetic vascular graft infection
Source: Front Med (Lausanne). 2023 May 19;10:1199657. doi: 10.3389/fmed.2023.1199657 (PMC10235614; doi:10.3389/fmed.2023.1199657)

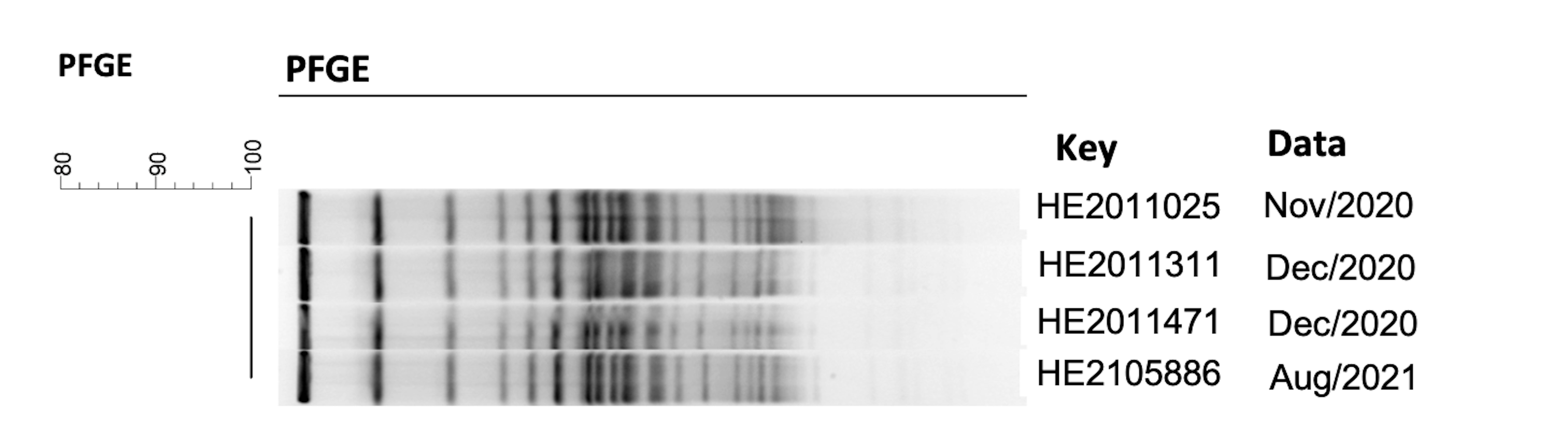

Supplement: Supplementary Figure S1 — PFGE (Pulsed Field Gel Electrophoresis) patterns of four sequential Pseudomonas aeruginosa isolates. [file Image_1.TIF]
