# Supplementary material for: Case report: Analysis of phage therapy failure in a patient with a Pseudomonas aeruginosa prosthetic vascular graft infection
Source: Front Med (Lausanne). 2023 May 19;10:1199657. doi: 10.3389/fmed.2023.1199657 (PMC10235614; doi:10.3389/fmed.2023.1199657)
Supplement: Supplementary Table S1 — Mutations encountered in proteins known to be related to antimicrobial resistance in Pseudomonas aeruginosa. The mutations present in P. aeruginosa isolate HE2105886 in comparison to P. aeruginosa isolate HE2011471 are shown. [file Data_Sheet_1.PDF]

Protein	Mechanism	Mutations[1]
AmpC	Structural change	**G27D, T105A, V205L, V356I**, **G391A**
AmpD	AmpC overexpression	None
AmpDh2	AmpC overexpression	**V40I**
AmpDh3	AmpC overexpression	None
AmpR	AmpC overexpression	**G283E, M288R**
ArmZ	MexXY overexpression	**C40R, L88P, S112N, D119E, I237V, V243A**
DacB	AmpC overexpression	None
DacC	PBP5	None
FtsI	PBP3	G8S
FusA1	Elongation factor G	H166D
GalU	Lipopolisacharide synthesis	None
GyrA	DNA gyrase (subunit A)	None
GyrB	DNA gyrase (subunit B)	S466F
MexA	Intrinsic antibiotic resistance	A111T
MexB	Intrinsic antibiotic resistance	**G957D, S1041E, V1042A**
MexC	MexCD overexpression	**E251Q,A262E,A277T,H310R,S330A,A378T,A384V**
MexD	MexCD overexpression	**T87S,S845A**
MexE	MexEF overexpression	None
MexF	MexEF overexpression	None
MexR	MexAB overexpression	M1_L13del, **V132A**
MexS	MexEF overexpression/OprD downregulation	**V73A, D249N**
MexT	MexEF overexpression/OprD downregulation	M1_A78del, **F172I**
MexX	Intrinsic antibiotic resistance	**A30T, K329Q, L331V, W358R**
MexY	Intrinsic antibiotic resistance	**I536V, T543A, G589A,Q840E, N1036T**
MexZ	MexXY overexpression	**G89S**
Mpl	Recycling of cell wall components (AmpC overexpression)	R73C
NalC	MexAB overexpression	**G71E, D79E, S209R**
NalD	MexAB overexpression	None
NfxB	MexCD overexpression	none (HE2011025,HE2040684)<br>multiple (HE2011311, HE2011471,HE2105886)[2]
OprD	Inactivation OprD	**T103S,K115T,F170L,P186G,V189T,R310E,A315G,G425A**
OprJ	MexCD overexpression	None
OprM	Intrinsic antibiotic resistance	None
OprN	MexEF overexpression	**S13P**
ParC	DNA topoisomerase IV (subunit A)	**P752T**
ParE	DNA topoisomerase IV (subunit B)	**D533E**
ParR	MexXY and MexEF overexpression/ OprD downregulation	**S170N,L153R**
ParS	MexXY overexpression/OprD downregulation	aa70InsPR, **H398R**
PbpA	PBP2	none
PmrA	Lipopolisacharide synthesis	none
PmrB	Lipopolisacharide synthesis	**S2P, A4T, V15I, G68S, Y345H**

[1]mutations described previously as natural polymorphisms are in bold type
[2]see multiple alignment sequences using Clustal (EMBL-EBI) in Figure S2
