# Supplementary material for: Case report: Analysis of phage therapy failure in a patient with a Pseudomonas aeruginosa prosthetic vascular graft infection
Source: Front Med (Lausanne). 2023 May 19;10:1199657. doi: 10.3389/fmed.2023.1199657 (PMC10235614; doi:10.3389/fmed.2023.1199657)
Supplement: Supplementary Table S2 — Mutations encountered in proteins probably to be related to phage resistance in response to lytic phage infection in P. aeruginosa isolates P. aeruginosa isolate HE2105886 in comparison to P. aeruginosa isolate HE2011471. a Proteins located in prophages as identified by Phaster and SourceFinder. [file Data_Sheet_2.PDF]

Protein	Function	Phage Infection (References)	SNP (HE2011471:HE2105886)	Aminoacid change (HE2011471:HE2105886)	Quorum Sensing (References)
**Receptors/Pilus/Regulators/Efflux pumps Transport: Antimicrobial susceptibility**					
FemA receptor	Siderophore transport Iron transport	Phage receptor (1)	G>T	A274D	(2)
TonB- dependent receptor	Antibiotic efflux. Iron transport	Phage receptor (3)	G>A	P679L	(2)
Filamentous haemagglutinin pilus	Surface adhesion	Phage receptor(4)	T>G; GGCCGGCAGTCTGCTC GGCGAGGCTCTGGACC TGCCCGCC>G; A>C; T>G; T>G	V1881G; del2874-2887; T2888P; L2890R; L2896R	(5)
Transcriptional regulator, AcrR family	Efflux pumps regulation	Phage receptor (6)	C>T	S36F	(7)
Magnesium and cobalt efflux CorC	Ion Resistance. Target of the bacteriocin	Phage receptor (8)	T>G	T213P	(9)
**Quorum Sensing activation**					
Acyl-homoserine lactone acylase PvdQ, quorum-quenching	Quorum sensing inhibition. Virulence	Phage Resistance (10)	GC	H695T; Q696S; L697Y; H698I; P699Q; T700L; G701V; D702T; L703F; R705E; del706708; Q709E; S711P; R712K;	(11)

				V713A; A714R; F716L; P718A; V719F; Q720S; R721Q; A723S	
**Prophages[a]: Resistance to Lytic Phage Infection**					
Zona occludens toxin	Filamentous Prophage assembly	Phage Resistance (12)	T>C	S244W; M249L; V251T; A254G; del255-280	(12)
Phage terminase, large subunit GpA	Prophage assembly	Phage Resistance (13)	Ins1-84; G>A	ins1-28; A355T	(12)
Prophage Clp protease	Prophage	Phage Resistance (14)	C>T	V134G	(15)
**Small molecules: Resistance to Lytic Phage Infection**					
SAM-dependent methyltransfere YafE	Aminoglycoside regulation/ Antibiotic Resistance	Phage Resistance (16)	C>T	V134G	(17)
**TA systems: Resistance to Lytic Phage Infection**					
Glutamyl-tRNA synthetase HipBA toxin-antitoxin	Persistence	Phage Resistance (18)	A>C	C432R	(19)
**Oxidative Stress/Secretion Systems/Virulence: Response to Lytic Phage Infection**					
Flavodoxin reductases	Tolerance to oxidative stress	Phage infection (20)	C>A	R39L	(21)
Catalase KatE		Phage infection (22)	C>T	R358H	(23)

T1SS secreted agglutinin RTX	Secretion Systems	Phage infection (24)	G>T	P695T	(25)
Transcriptional anti-antiactivator ExsC T3SS		Phage infection (26)	C>T	G66E	(25)
VgrG protein Effector of the T6SS		Phage infection (27)	T>C	L32P	(25)
VgrG protein Effector of the T6SS		Phage infection (27)	A>G	V43M	(25)
Pyoverdine synthetase PvdL	Virulence	Phage infection (28)	T>G	D1938A; L2241V; L2243M; L2247I; G2252A; L2254M; L2266V; L2272E; L2275Q; Q2276A; I2279M	(29)
Cis/trans isomerase		Virulence; resistance to cell wall solvents. (30)	G>T	P82T	(31)
Isocitrate dehydrogenase [NADP]		Glyoxylate shunt linked with virulence. (32)	A>C	T474P	(33)
MOSC domain-containing protein		Molibdenum cofactor. Biosynthesis of Moco (33)	T>G	V25G	(34)
GTP 3',8-cyclase		Biosynthesis of Moco	A>C	T257P	(34)

Molibdenum		(35)			
Phosphate synthase II PhzC (chorismate)		Phenazine biosynthesis. (36)	del765-834	del255-278	(37)

REFERENCES

1. Sundarrajan S, Raghupatil J, Vipra A, Narasimhaswamy N, Saravanan S, Appaiah C, et al. Bacteriophage-derived CHAP domain protein, P128, kills *Staphylococcus* cells by cleaving interpeptide cross-bridge of peptidoglycan. Microbiology (Reading). 2014;160(Pt 10):2157-69.
2. Cornelis P, Matthijs S, Van Oeffelen L. Iron uptake regulation in *Pseudomonas aeruginosa*. Biometals. 2009;22(1):15-22.
3. Rabsch W, Ma L, Wiley G, Najar FZ, Kaserer W, Schuerch DW, et al. FepA- and TonB-dependent bacteriophage H8: receptor binding and genomic sequence. J Bacteriol. 2007;189(15):5658-74.
4. Jouravleva EA, McDonald GA, Marsh JW, Taylor RK, Boesman-Finkelstein M, Finkelstein RA. The *Vibrio cholerae* mannose-sensitive hemagglutinin is the receptor for a filamentous bacteriophage from *V. cholerae* O139. Infect Immun. 1998;66(6):2535-9.
5. Sun J, Li X, Qiu Y, Xue X, Zhang M, Yang W, et al. Quorum sensing regulates transcription of the pilin gene mshA1 of MSHA pilus in *Vibrio parahaemolyticus*. Gene. 2022;807:145961.
6. Nazarov PA. MDR Pumps as Crossroads of Resistance: Antibiotics and Bacteriophages. Antibiotics (Basel). 2022;11(6).
7. Chin-A-Woeng TF, van den Broek D, Lugtenberg BJ, Bloemberg GV. The *Pseudomonas chlororaphis* PCL1391 sigma regulator psrA represses the production of the antifungal metabolite phenazine-1-carboxamide. Mol Plant Microbe Interact. 2005;18(3):244-53.
8. Bohm K, Porwollik S, Chu W, Dover JA, Gilcrease EB, Casjens SR, et al. Genes affecting progression of bacteriophage P22 infection in *Salmonella* identified by transposon and single gene deletion screens. Mol Microbiol. 2018;108(3):288-305.
9. Hounmanou YMG, Leekitcharoenphon P, Hendriksen RS, Dougnon TV, Mdegela RH, Olsen JE, et al. Surveillance and Genomics of Toxigenic *Vibrio cholerae* O1 From Fish, Phytoplankton and Water in Lake Victoria, Tanzania. Front Microbiol. 2019;10:901.
10. León-Félix J, Villicaña C. The impact of quorum sensing on the modulation of phage-host interactions. J Bacteriol. 2021.

11. Utari PD, Setroikromo R, Melgert BN, Quax WJ. PvdQ Quorum Quenching Acylase Attenuates *Pseudomonas aeruginosa* Virulence in a Mouse Model of Pulmonary Infection Front Cell Infect Microbiol. 2018;8:119.

12. Ambroa A, Blasco L, López-Causapé C, Trastoy R, Fernandez-García L, Bleriot I, et al. Temperate Bacteriophages (Prophages) in *Pseudomonas aeruginosa* Isolates Belonging to the International Cystic Fibrosis Clone (CC274). . Front Microbiol. 2020;11:556706.

13. Owen SV, Wenner N, Dulberger CL, Rodwell EV, Bowers-Barnard A, Quinones-Olvera N, et al. Prophages encode phage-defense systems with cognate self-immunity. Cell Host Microbe. 2021;29(11):1620-33.e8.

14. Geuskens V, Mhammedi-Alaoui A, Desmet L, Toussaint A. Virulence in bacteriophage Mu: a case of trans-dominant proteolysis by the *Escherichia coli* Clp serine protease. EMBO J. 1992;11(13):5121-7.

15. Yang N, Lan L. *Pseudomonas aeruginosa* Lon and ClpXP proteases: roles in linking carbon catabolite repression system with quorum-sensing system. Curr Genet. 2016;62(1):1-6.

16. Hardy A, Kever L, Frunzke J. Antiphage small molecules produced by bacteria - beyond protein-mediated defenses. Trends Microbiol. 2023;31(1):92-106.

17. Kang Y, Kim H, Goo E, Jeong H, An JH, Hwang I. Unraveling the role of quorum sensing-dependent metabolic homeostasis of the activated methyl cycle in a cooperative population of *Burkholderia glumae*. Sci Rep. 2019;9(1):11038.

18. LeRoux M, Laub MT. Toxin-Antitoxin Systems as Phage Defense Elements. Annu Rev Microbiol. 2022;76:21-43.

19. Hemati S, Azizi-Jalilian F, Pakzad I, Taherikalani M, Maleki A, Karimi S, et al. The correlation between the presence of quorum sensing, toxin-antitoxin system genes and MIC values with ability of biofilm formation in clinical isolates of *Pseudomonas aeruginosa*. Iran J Microbiol. 2014;6(3):133-9.

20. Lamb DC, Follmer AH, Goldstone JV, Nelson DR, Warrilow AG, Price CL, et al. On the occurrence of cytochrome P450 in viruses. Proc Natl Acad Sci U S A. 2019;116(25):12343-52.

21. Moyano AJ, Tobares RA, Rizzi YS, Krapp AR, Mondotte JA, Bocco JL, et al. A long-chain flavodoxin protects *Pseudomonas aeruginosa* from oxidative stress and host bacterial clearance. PLoS Genet. 2014;10(2):e1004163.

22. Mancini S, Imlay JA. The induction of two biosynthetic enzymes helps *Escherichia coli* sustain heme synthesis and activate catalase during hydrogen peroxide stress. Mol Microbiol. 2015;96(4):744-63.
23. Liao H, Zhong X, Xu L, Ma Q, Wang Y, Cai Y, et al. Quorum-sensing systems trigger catalase expression to reverse the oxyR deletion-mediated VBNC state in *Salmonella typhimurium.* Res Microbiol. 2019;170(2):65-73.
24. Zhou W, Li Y, Li Z, Ma B, Jiang X, Hu C, et al. Genomic Changes and Genetic Divergence of. Front Microbiol. 2021;12:710262.
25. Pena RT, Blasco L, Ambroa A, Gonzalez-Pedrajo B, Fernandez-Garcia L, Lopez M, et al. Relationship Between Quorum Sensing and Secretion Systems. Front Microbiol. 2019;10:1100.
26. Nakamura K, Ogura Y, Gotoh Y, Hayashi T. Prophages integrating into prophages: A mechanism to accumulate type III secretion effector genes and duplicate Shiga toxin-encoding prophages in *Escherichia coli.* PLoS Pathog. 2021;17(4):e1009073.
27. Navarro-Garcia F, Ruiz-Perez F, Cataldi Á, Larzábal M. Type VI Secretion System in Pathogenic *Yersinia*. Front Microbiol. 2019;10:1965.
28. Hosseinidoust Z, Tufenkji N, van de Ven TG. Predation in homogeneous and heterogeneous phage environments affects virulence determinants of *Pseudomonas aeruginosa*. Appl Environ Microbiol. 2013;79(9):2862-71.
29. Y Ramírez-Rueda R, Salvador MJ. Phenotypic detection of quorum sensing inhibition in *Pseudomonas aeruginosa* pyoverdine and swarming by volatile organic products. Future Microbiol. 2020;15:1147-56.
30. Bothmann H, Pluckthun A. The periplasmic *Escherichia coli* peptidylprolyl cis,trans-isomerase FkpA. I. Increased functional expression of antibody fragments with and without cis-prolines. J Biol Chem. 2000;275(22):17100-5.
31. Ünal CM, Steinert M. Microbial peptidyl-prolyl cis/trans isomerases (PPIases): virulence factors and potential alternative drug targets. Microbiol Mol Biol Rev. 2014;78(3):544-71.
32. Wang FS, Whittam TS, Selander RK. Evolutionary genetics of the isocitrate dehydrogenase gene (icd) in *Escherichia coli* and *Salmonella enterica.* J Bacteriol. 1997;179(21):6551-9.

33. Yan C, Li X, Zhang G, Zhu Y, Bi J, Hao H, et al. Quorum Sensing-Mediated and Growth Phase-Dependent Regulation of Metabolic Pathways in *Hafnia alvei* H4. Front Microbiol. 2021;12:567942.
34. Filiatrault MJ, Tombline G, Wagner VE, Van Alst N, Rumbaugh K, Sokol P, et al. *Pseudomonas aeruginosa* PA1006, which plays a role in molybdenum homeostasis, is required for nitrate utilization, biofilm formation, and virulence. PLoS One. 2013;8(2):e55594.
35. Shanmugam KT, Stewart V, Gunsalus RP, Boxer DH, Cole JA, Chippaux M, et al. Proposed nomenclature for the genes involved in molybdenum metabolism in *Escherichia coli* and *Salmonella typhimurium*. Mol Microbiol. 1992;6(22):3452-4.
36. Chen Y, Shen X, Peng H, Hu H, Wang W, Zhang X. Comparative genomic analysis and phenazine production of *Pseudomonas chlororaphis*, a plant growth-promoting rhizobacterium. Genom Data. 2015;4:33-42.
37. Wang Z, Huang X, Jan M, Kong D, Wang W, Zhang X. Lon protease downregulates phenazine-1-carboxamide biosynthesis by degrading the quorum sensing signal synthase PhzI and exhibits negative feedback regulation of Lon itself in *Pseudomonas chlororaphis* HT66. Mol Microbiol. 2021;116(2):690-706.
